# Prediction of bone invasion of oral squamous cell carcinoma using a magnetic resonance imaging-based machine learning model

**DOI:** 10.1007/s00405-024-08862-z

**Published:** 2024-07-31

**Authors:** Elif Meltem Aslan Öztürk, Gürkan Ünsal, Ferhat Erişir, Kaan Orhan

**Affiliations:** 1 Department of Dentomaxillofacial Radiology, Faculty of Dentistry, Lokman Hekim University, Ankara, Turkey; 2 Schulich School of Medicine and Dentistry, Western University, London, ON Canada; 3 Department of Otorhinolaryngology and Head and Neck Surgery, Faculty of Medicine, Near East University, Kyrenia, Cyprus; 4 Department of Dentomaxillofacial Radiology, Faculty of Dentistry, Ankara University, Ankara, Turkey

**Keywords:** Machine learning, Oral squamous cell carcinoma, Bone invasion, Magnetic resonance imaging

## Abstract

**Objectives:**

Radiomics, a recently developed image-processing technology, holds potential in medical diagnostics. This study aimed to propose a machine-learning (ML) model and evaluate its effectiveness in detecting oral squamous cell carcinoma (OSCC) and predicting bone metastasis using magnetic resonance imaging (MRI).

**Materials-methods:**

MRI radiomic features were extracted and analyzed to identify malignant lesions. A total of 86 patients (44 with benign lesions without bone invasion and 42 with malignant lesions with bone invasion) were included. Data and clinical information were managed using the RadCloud Platform (Huiying Medical Technology Co., Ltd., Beijing, China). The study employed a hand-crafted radiomics model, with the dataset randomly split into training and validation sets in an 8:2 ratio using 815 random seeds.

**Results:**

The results revealed that the ML method support vector machine (SVM) performed best for detecting bone invasion (AUC = 0.999) in the test set. Radiomics tumor features derived from MRI are useful to predicting bone invasion from oral squamous cell carcinoma with high accuracy.

**Conclusions:**

This study introduces an ML model utilizing SVM and radiomics to predict bone invasion in OSCC. Despite the promising results, the small sample size necessitates larger multicenter studies to validate and expand these findings.

## Introduction

Oral squamous cell carcinoma (OSCC) accounts approximately 5% of all malignant tumors [1, 2] and is primarily linked to tobacco and alcohol consumption [3, 4]. The International Agency for Research on Cancer (IARC) has identified additional carcinogens such as betel with or without tobacco, HPV 16, and passive smoking with varying degrees of evidence [3, 5].

Diagnosing OSCC often occurs in advanced stages due to its rapid growth despite the absence of initial clinical symptoms [6]. To assess tumor characteristics, radiological examinations, including computed tomography (CT) or magnetic resonance imaging (MRI), become crucial for evaluating size, depth, and potential bone tissue invasion [7, 8]. Treatment involves surgical intervention, adjuvant radiation, or combined radiotherapy/chemotherapy, determined by the tumor’s stage [9].

Bone invasion in the maxillary and mandibular bones is common in oral squamous cell carcinoma (OSCC). Cortical or medullary bone tissue invasion classifies the tumor as stage IVa according to the TNM classification system [10]. Detecting bone invasion in OSCC significantly impacts the patient’s prognosis and is crucial for surgical planning and determining the necessity of adjuvant therapy [11]. In cases of OSCC with bone invasion, the 5-year survival rate is approximately 50%, with surgical resection yielding a 47% survival rate and chemotherapy yielding a 56% survival rate [12, 13].

CT and MRI are both effective for detecting bone invasion or erosion in OSCC. These imaging methods exhibit similar sensitivity, specificity, and accuracy. Literature reports sensitivity, specificity, and accuracy values ranging from 41.7 to 89%, 86.9–100%, and 71.2–85% for CT, and 58.3-95.24%, 73–100%, and 75.8–93% for MRI, respectively [10, 14]. However, in clinical practice, MRI is often preferred for imaging head and neck tumors due to its superior soft tissue contrast [15]. MRI examinations of OSCC typically utilize sequences such as T1-weighted turbo spin echo (T1-SE/TSE), T2-weighted turbo spin echo/fast spin echo (T2-TSE/FSE), T1-weighted fat-saturated (T1-FS), T2-weighted fat-saturated (T2-FS), and diffusion-weighted imaging (DWI) pulse sequences [15].

While radiological images alone provide limited information about tissue complexity and heterogeneity, radiomics analysis can significantly enhance the understanding of disease prognosis and treatment response. By modeling quantities obtained from radiomics with clinical and laboratory parameters, it is possible to determine survival rates, tumor staging, lymph node metastasis, distant metastasis, total tumor burden, biomarker detection, treatment response, side effects, tumor heterogeneity, prognosis, and recurrence [16–18]. Radiomics, relying heavily on machine learning (ML) models, enables a comprehensive assessment of the biological properties of lesions captured in medical images [17, 19].

In the literature, studies are using MRI-based radiomics for the diagnosis of local invasion, metastasis, prognosis, and perineural invasion in MRI images of head and neck malignancies in various anatomical regions, including OSCC [15, 20–25]. This study aims to propose a ML model using radiomic analysis of MRI images to detect OSCC and predict potential bone metastasis.

## Materials and methods

The retrospective analysis of anonymized data was ethically approved by the Research Ethics Committee prior to the commencement of the study (Protocol No: 2024051).

### Study population

A cohort comprising 86 patients (42 females and 45 males; mean age: 58.3) within the age range of 45 to 74 years was enrolled in the study. MRI images from these patients, classified into 44 benign cases without bone invasion and 42 malignant cases with bone invasion, were evaluated.

The inclusion criteria were as follows:

- Age over 18 years.
- Histologically confirmed diagnosis of OSCC.
- Absence of previous bone operations.
- Performance of a preoperative MRI scan.

The exclusion criteria were as follows:

- Presence of artifacts that significantly affect MRI evaluations.
- Preexisting osteoradionecrosis.
- Preexisting drug-related osteonecrosis of the jaw.
- Presence of malignancies other than OSCC.
- Lack of histological examination or incomplete follow-up.

### MRI imaging procedure

All patients underwent imaging utilizing a 1.5-T MRI scanner (Signa HDxt, GE, Milwaukee, USA) equipped with a head and neck coil. Conventional T1- and T2-weighted FSE images were acquired with the following parameters: TR = 450-2500-3000 ms, TE = 10–70 milliseconds, echo train length (ETL) = 10, matrix size of 256 × 256, slice thickness of 3 mm.

DWI axial images were obtained with the following parameters: TR = 8400 ms, TE = 17 ms, number of signal intensity acquisitions, using the single-shot spin-echo, echo-planar imaging technique, with an FOV of 200 mm, matrix size of 120 × 120, section thickness of 3 mm, section gap of 0.3 mm, and b-values of 0 and 1000 s/mm².

Axial and coronal contrast-enhanced T1-weighted images (CET1W) were obtained with the following parameters: TR/TE = 550 ms/10 ms, flip angle = 90°, FOV = 15 × 15 cm, matrix size = 320 × 256, slice thickness = 3.5 mm, gap = 0.3 mm, NEX = 2. Additionally, coronal CET1W was acquired with the following parameters: TR/TE1/TE2=(shortest) 6 ms/(shortest) 2 ms/(shortest) 3 ms, flip angle = 15°, slice thickness = 1.1 mm.

### Data management

Radiomics is an emerging field that converts imaging data into a high dimensional mineable feature space using a large number of automatically extracted data-characterization algorithms. Thus, the Radcloud platform (Huiying Medical Technology Co., Ltd., Beijing, China) was used to manage imaging data, clinical data, and subsequent radiomics statistics analysis. These radiomics platforms have the potential to uncover the distinctive imaging algorithms to quantify the state of diseases, and thereby provide valuable information for personalized medicine. Moreover, they can measure features in an imaging exam that include intensity, shape, texture, wavelet, and LOG features, etc. to build predictive or prognostic non-invasive biomarkers or imaging modalities. This platform can be used for the extraction of Radiomics features from 2D and 3D images and binary masks on different imaging modalities such as CT and MRI.

The radiomics platform was utilized to manage both imaging and clinical data, facilitating subsequent radiomics statistical analysis. Radiomics platforms have the potential to reveal different imaging algorithms to measure the status of diseases and thus provide valuable information for personalized medicine. The separation of training and validation datasets was randomized using an (8:2) ratio and 815 random seeds.

### Imaging segmentation

**Fig. 1:**
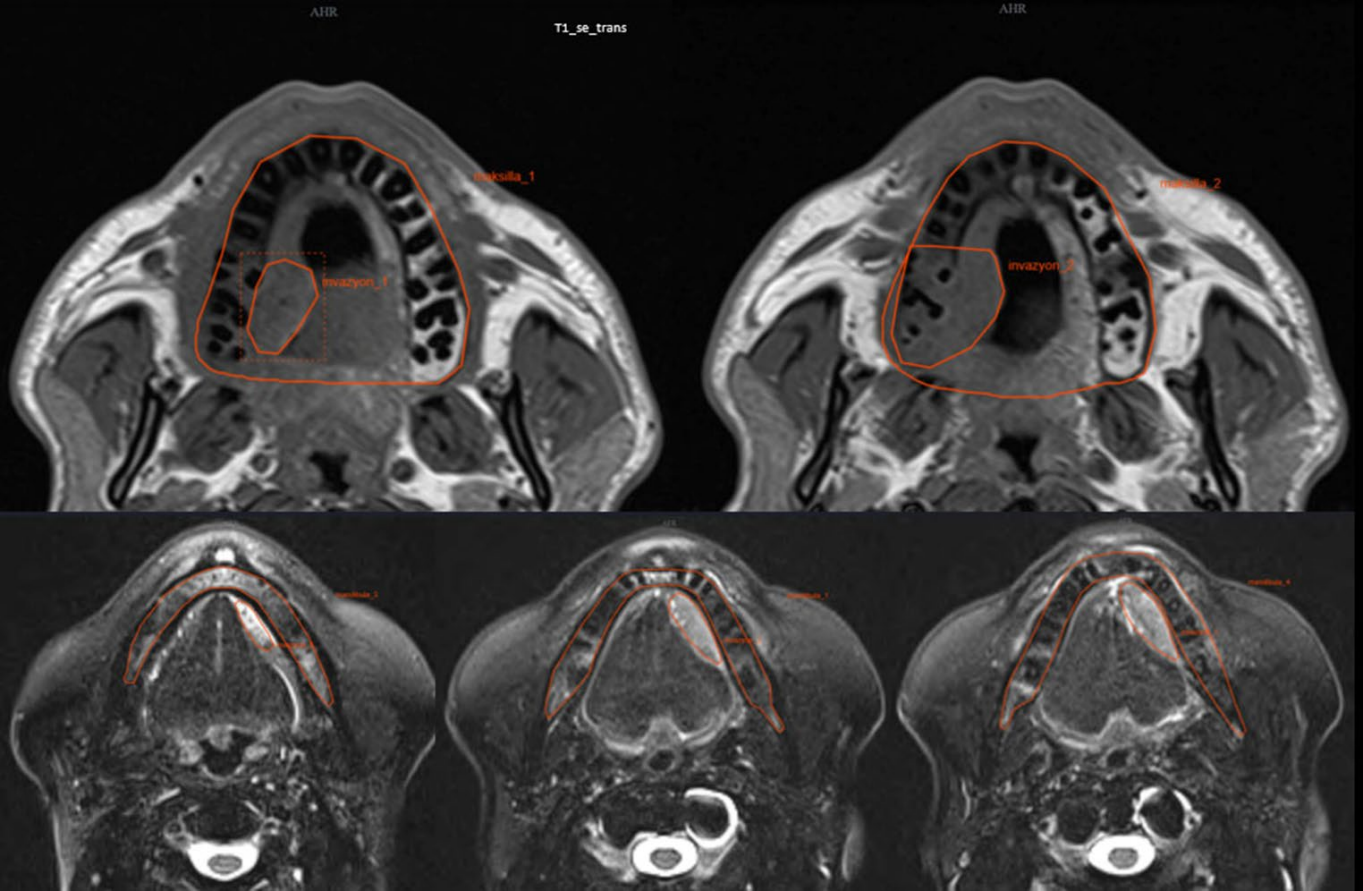
T1-Se axial MR images showing bone invasion area of the maxillary and mandibular bone and oral SCC on T2-TSE-transversal-fat-suppressed MRI images which was manually defined on consecutive MR slices

**Fig. 2:**
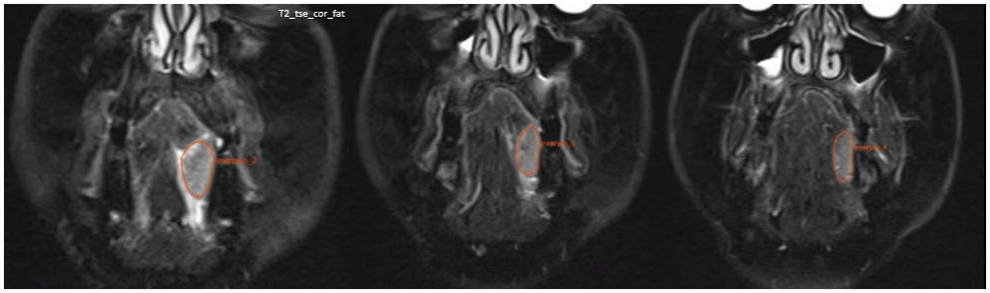
T2-TSE-coronal-fat-suppressed MRI images showing the oral SCC with manual delineation on consecutive MR slices

Maxilla, mandible, and bone invasion areas were manually defined on the MRI images independently by the specialist radiologist and senior specialist radiologist (EMAO and KO) blinded to the patient’s clinical information. The software allows contours to be drawn using a LASSO tool that can draw a manually shaped area defined by the mouse. When the tool moves, it can select objects within the defined contours, allowing boundaries to be adjusted. All contours were then reviewed again and evaluated together for final adjustments by consensus. In the case of oral SCC with bone invasion, the MRI image of the mandibular bone and invasion area were manually defined as shown in Figs. 1 and 2.

### Feature extraction and selection

A total of 247 radiomic features were identified from the VOIs of MRI images using the Radiomics platform. These radiomics features were under first-order, shape, and texture classifications. In particular, texture features including gray level co-occurrence matrix (GLCM), gray level run length matrix (GLRLM), and gray level size zone matrix (GLSZM) were utilized (Table 1). Additionally, intensity and texture features were calculated on the original image and derived images obtained by applying various filters such as exponential, logarithm, square, square root, and wavelet (wavelet-LHL, wavelet-L, wavelet-HLL, wavelet-LLH, wavelet-HLH, wavelet-HHH, wavelet-HHL, wavelet-LLL). The features correspond to the definitions defined by the Imaging Biomarker Standardization Initiative (IBSI). To decrease the dimensionality of the features, variance thresholding methods were used to incrementally select the most relevant features. A variance threshold was also applied to reduce features (variance threshold = 0.8).

**Table 1:** Radiomic features selected for quantifying the heterogeneity differences

Radiomic group	Associated filter	No. of features	Radiomic features
First-order statistics	None	26	Energy, total energy, entropy, minimum, 10 percentile, 90 percentile, maximum, mean, median, interquartile range, range, mean absolute deviation, robust mean absolute deviation, root mean square, standard deviation, skewness, kurtosis, variance
Shape	None	14	Volume, surface area, surface volume ratio, spherical disproportion, maximum 3D diameter, maximum 2d diameter column, maximum 2d diameter row, elongation
Texture features	GLCM	207	Autocorrelation, average intensity, cluster prominence, cluster shade, cluster tendency, contrast, difference average, difference entropy, difference variance, dissimilarity, entropy, sum average, sum entropy, sum variance, sum squares
Texture features	GLSZM		Large area emphasis, gray level non uniformity, size zone non uniformity, gray level variance, zone entropy, high gray level zone emphasis, small area high gray level emphasis,, large area high gray level emphasis
Texture features	GLRLM		Gray level non uniformity, run length non uniformity, gray level variance, run entropy, high gray level run emphasis, short run high gray level emphasis, long run high level emphasis

Label: GLCM = Gray-level Co-occurrence Matrix, GLSZM = Gray-Level Size Zone Matrix, GLRLM = Gray Level Run Length Matrix

### Consensus clustering

A consensus clustering was used to cluster the radiomic features extracted from the training sets in the maxilla, mandible, and bone invasion areas.

Consensus clustering aims to find a single partitioning of the data from multiple existing underlying partitions. Many algorithms have been suggested in the literature to address computational challenges, such as co-association matrix-based methods, graph-based methods, prototype-based approaches, and other heuristic methods [26–28].

The k-means clustering method, the most proposed here, is of particular interest due to its simplicity and high efficiency. K-means clustering is one of the most widely used non-supervised machine learning algorithms for categorizing a given dataset into k-groups of clusters, where k denotes the number of groups predetermined by observers. Within k-means clustering, each cluster is defined by its center, which corresponds to the average of the points that are assigned to the cluster [29, 30].

The first step in constructing consensus clustering is to construct an n × n “consensus matrix” which is based on the input clustering results. Thereby, an estimate is performed for the interval of the appropriate number of clusters utilizing k-means clustering. Cluster consensus is described as the average consensus between all pairs of features belonging to the same cluster. The cluster agreement should be between (1,-1), where this value indicates the stability of a cluster over resampling.

Cluster stability is defined as follows:

- Consensus < 0.5, weak stability.
- 0.5 ≤ consensus < 0.75, moderate stability.
- Consensus ≥ 0.75, high stability.

Consensus clustering was implemented using the Radcloud platform (Huiying Medical Technology Co., Ltd., Beijing, China).

### Statistical analysis

All statistical analyses were conducted on the Radcloud platform (Huiying Medical Technology Co., Ltd., Beijing, China). Computer-generated random numbers were used to assign 80% of VOIs to the training dataset and 20% to the validation dataset. Six classifiers, which include logistic regression (LR), random forest (RF), decision tree (DT), k-nearest neighbors (KNN), XGBoost, and support vector machine (SVM), were used to build models that can predict bone invasion.

The performance of the models was assessed by sensitivity, specificity, and ROC curves. The optimal cut-off value was defined as the point at which the sensitivity plus specificity was maximized. Both the area under the curve (AUC) and prediction accuracy were measured for both training and validation sets. Subsequently, we employed four indicators to evaluate the performance of the classifiers, which included P (sensitivity = true positives/(true positives + false positives)), R (recall = true positives/(true positives + false negatives)), F1-score (F1 -score = P∗R∗2/(P + R)) and support (total number in the test set).

## Results

**Fig. 3:**
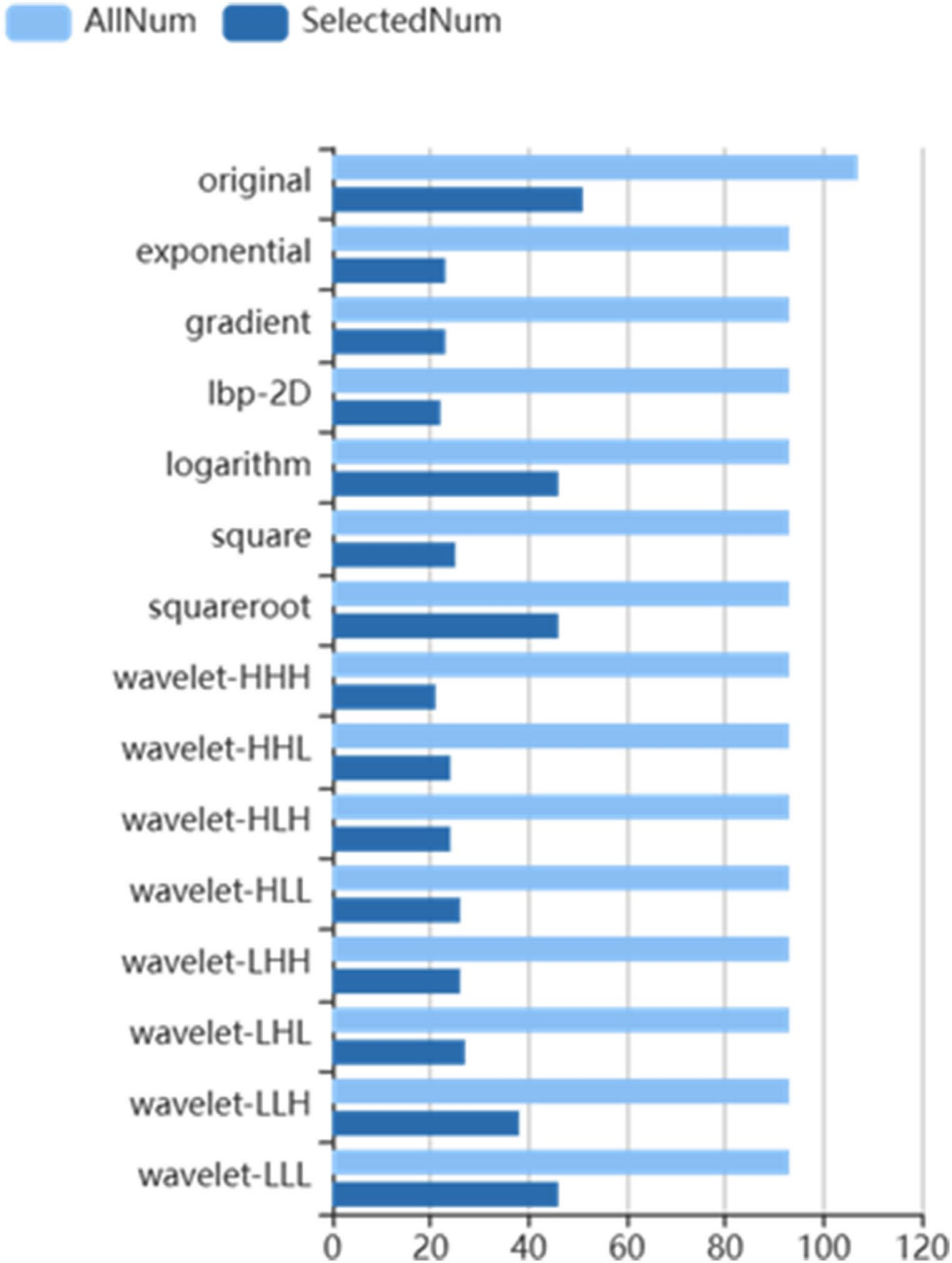
Variance threshold on feature selected. The variance threshold method was used to select radiomics features (variance threshold = 0.8), we selected 468 features from 1409 features

**Fig. 4:**
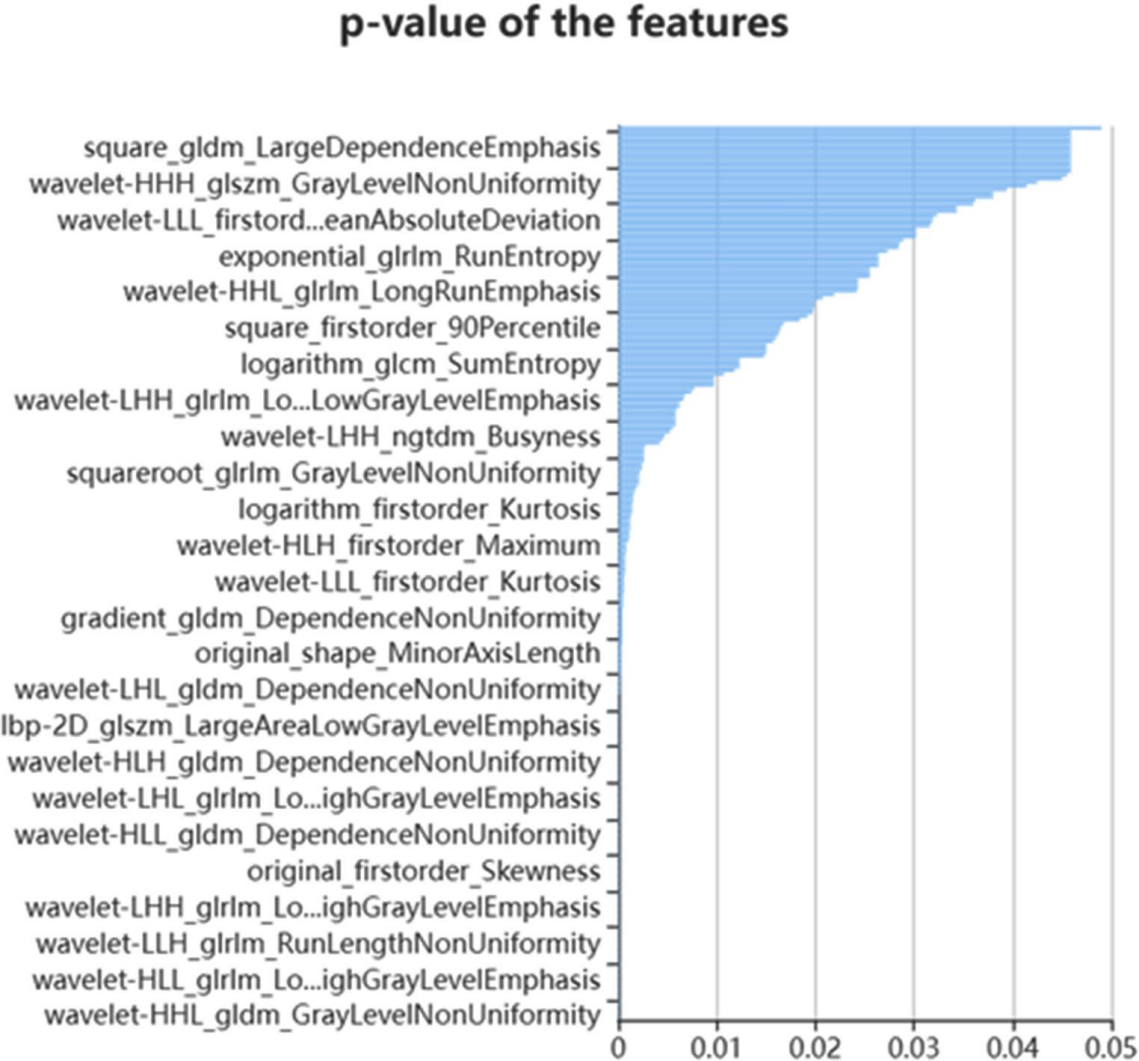
Select K-best method was used to further select the radiomics features; 25 features were chosen

**Fig. 5:**
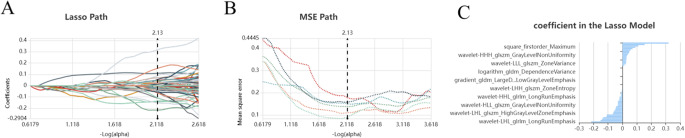
Lasso algorithm for feature selection: (**A**) Lasso path, (**B**) MSE path, and (**C**) coefficients in the Lasso model. The Lasso model was used to select four features corresponding to the optimal alpha value. Four features were selected

Out of a total of 1409 features, 468 were identified using the variance thresholding technique (Fig. 3), and the SelectKBest method was employed to select 25 features (Fig. 4). The LASSO algorithm was then utilized to identify the ten optimal features (Fig. 5).

The selected radiomic features were categorized into three groups: Group 1 (first-order statistics) comprised 58 commonly used predictors that quantitatively describe voxel intensity distribution within the image through basic metrics. Group 2 (shape and size-based features) included 8 two- and three-dimensional features capturing region shape and size, while the 12 textural features quantified heterogeneity differences derived from gray-level run length and gray-level co-occurrence texture matrices. Subsequently, an mRMR algorithm was applied, resulting in the retention of ten features further refined by the LASSO algorithm.

**Fig. 6:**
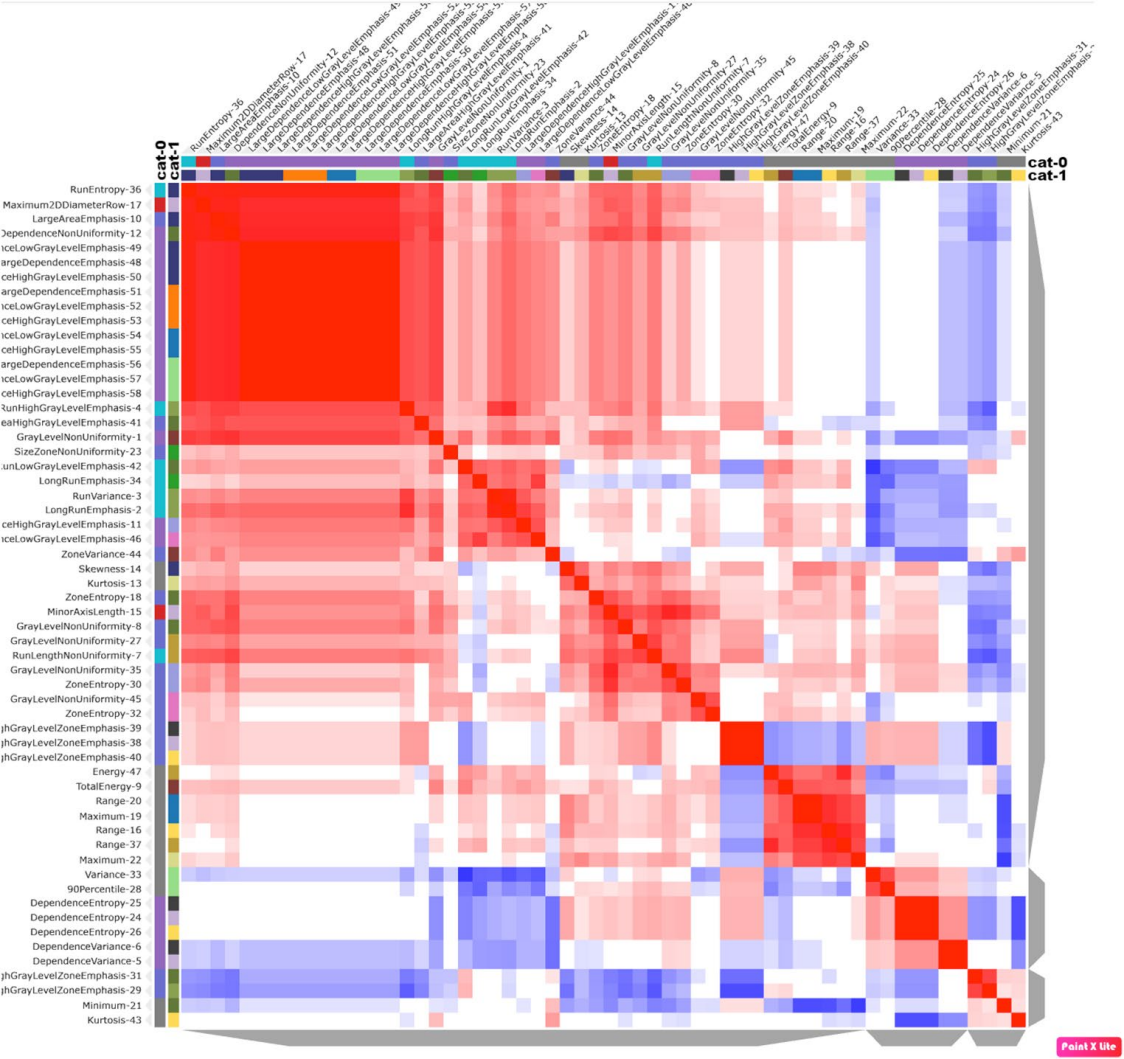
Depicts the confusion matrix after selecting radiomic features in the cohorts of groups

Figure 6 demonstrates the confusion matrix after the selection of radiomic features in the group cohorts, showcasing their initially significant prognostic performance (CI > 0.5) in distinguishing between the presence and absence of bone invasion (groups 0–1).

**Fig. 7:**
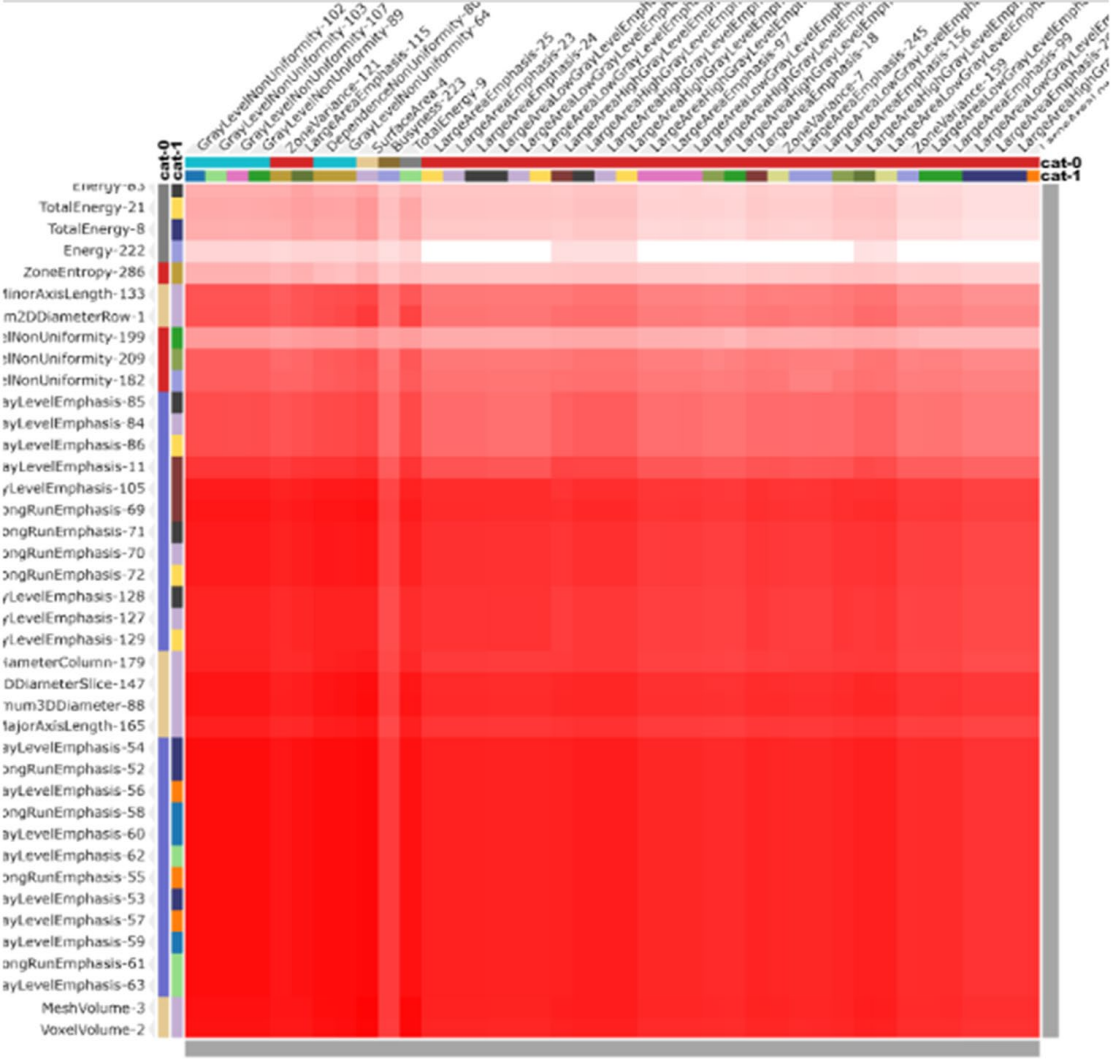
It demonstrates the cluster consensus maps of radiomic features for benign without bone invasion (0) and malignant with bone invasion (1) groups

**Fig. 8:**
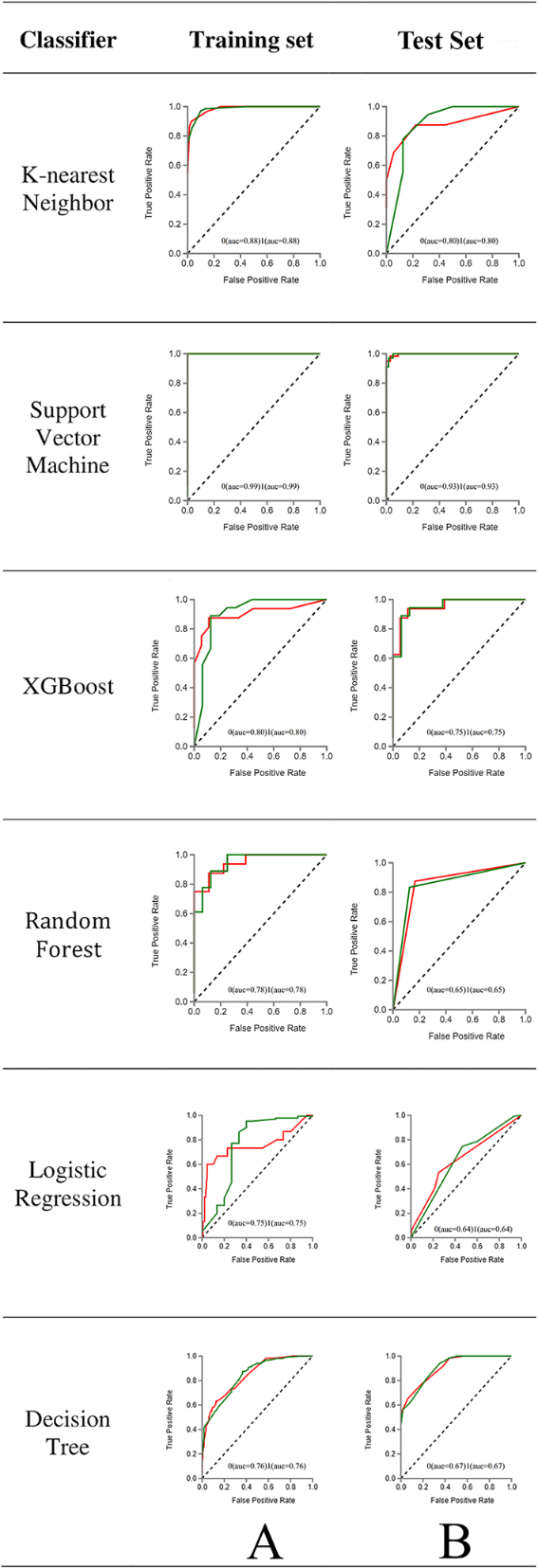
ROC curves of machine learning methods for classification. Green shows non-defective and red indicates defects. (A) ROC curve of the training dataset, (B) ROC curve of the test dataset

Figure 7 displays cluster consensus maps of radiomic features for benign (0) without bone invasion and malignant (1) with bone invasion groups. Tables 2 and 3 present the AUC sensitivity and specificity of the six classifiers in the training and test sets for detecting bone invasion of OSCC. The SVM classifier emerged as the most suitable method in both training and test sets, showcasing high diagnostic accuracy across four indicators. ROC curve analysis results for the training and validation sets are depicted in Fig. 8. For the selection of radiomic features, the SVM machine learning method exhibited high AUC values of 0.999 and 0.934 for the training and test sets, respectively. Tables 4 and 5 summarize four indicators (precision, recall, F1-score, support) for the six classifiers in detecting bone invasion of OSCC.

**Table 2:** ROC results with KNN, SVM, XGBoost, RF, LR and DT classifiers of training set

Classifiers	Category	AUC	95% CI	Sensitivity	Specificity
KNN	0	0.884	0.84–1.00	0.80	0.87
	1	0.884	0.84–1.00	0.80	0.87
SVM	0	0.999	0.97–1.00	0.98	0.97
	1	0.999	0.97–1.00	0.97	0.98
XGBoost	0	0.808	0.75–1.00	0.69	0.84
	1	0.808	0.75–1.00	0.84	0.69
RF	0	0.784	0.71–1.00	0.74	0.78
	1	0.784	0.71–1.00	0.78	0.74
LR	0	0.753	0.72–1.00	0.71	0.79
	1	0.753	0.72–1.00	0.79	0.71
DT	0	0.763	0.68–1.00	0.61	0.89
	1	0.763	0.68–1.00	0.89	0.61

**Table 3:** ROC results KNN, SVM, XGBoost, RF, LR and DT classifiers of test set

Classifiers	Category	AUC	95% CI	Sensitivity	Specificity
KNN	0	0.802	0.75–1.00	0.79	0.92
	1	0.800	0.75–1.00	0.92	0.79
SVM	0	0.934	0.81–1.00	0.94	0.78
	1	0.934	0.81–1.00	0.78	0.94
XGBoost	0	0.755	0.73–1.00	0.72	1
	1	0.755	0.73–1.00	1.00	0.72
RF	0	0.653	0.58–1.00	0.61	0.79
	1	0.653	0.58–1.00	0.79	0.61
LR	0	0.648	0.63–1.00	0.58	0.78
	1	0.648	0.63–1.00	0.78	0.58
DT	0	0.674	0.53–0.98	0.58	0.83
	1	0.674	0.63–0.98	0.83	0.58

**Table 4:** The results of four indicators -Precision, Recall, F1-score, support in training set

	Indicators	KNN	SVM	XGBoost	RF	LR	DT
0	Precision	0.92	1.00	0.79	0.77	0.78	0.72
	Recall	0.69	0.62	0.94	0.81	0.88	0.88
	F1-score	0.79	0.77	0.86	0.84	0.88	0.85
	Support	16	16	16	16	16	16
1	Precision	0.77	0.95	0.93	0.84	0.89	0.88
	Recall	0.94	1.00	0.78	0.89	0.89	0.83
	F1-score	0.85	0.86	0.85	0.86	0.89	0.86
	Support	18	18	18	18	18	18

**Table 5:** The results of four indicators -Precision, Recall, F1-score, support in test set

	Indicators	KNN	SVM	XGBoost	RF	LR	DT
0	Precision	0.86	0.97	0.76	0.67	0.79	0.70
	Recall	0.80	0.98	0.70	0.80	0.80	0.60
	F1-score	0.83	0.98	0.72	0.73	0.74	0.67
	Support	60	60	60	60	60	60
1	Precision	0.82	0.99	0.72	0.60	0.75	0.36
	Recall	0.87	0.97	0.74	0.73	0.76	0.77
	F1-score	0.84	0.98	0.77	0.70	0.70	0.74
	Support	68	68	68	68	68	68

**Fig. 9:**
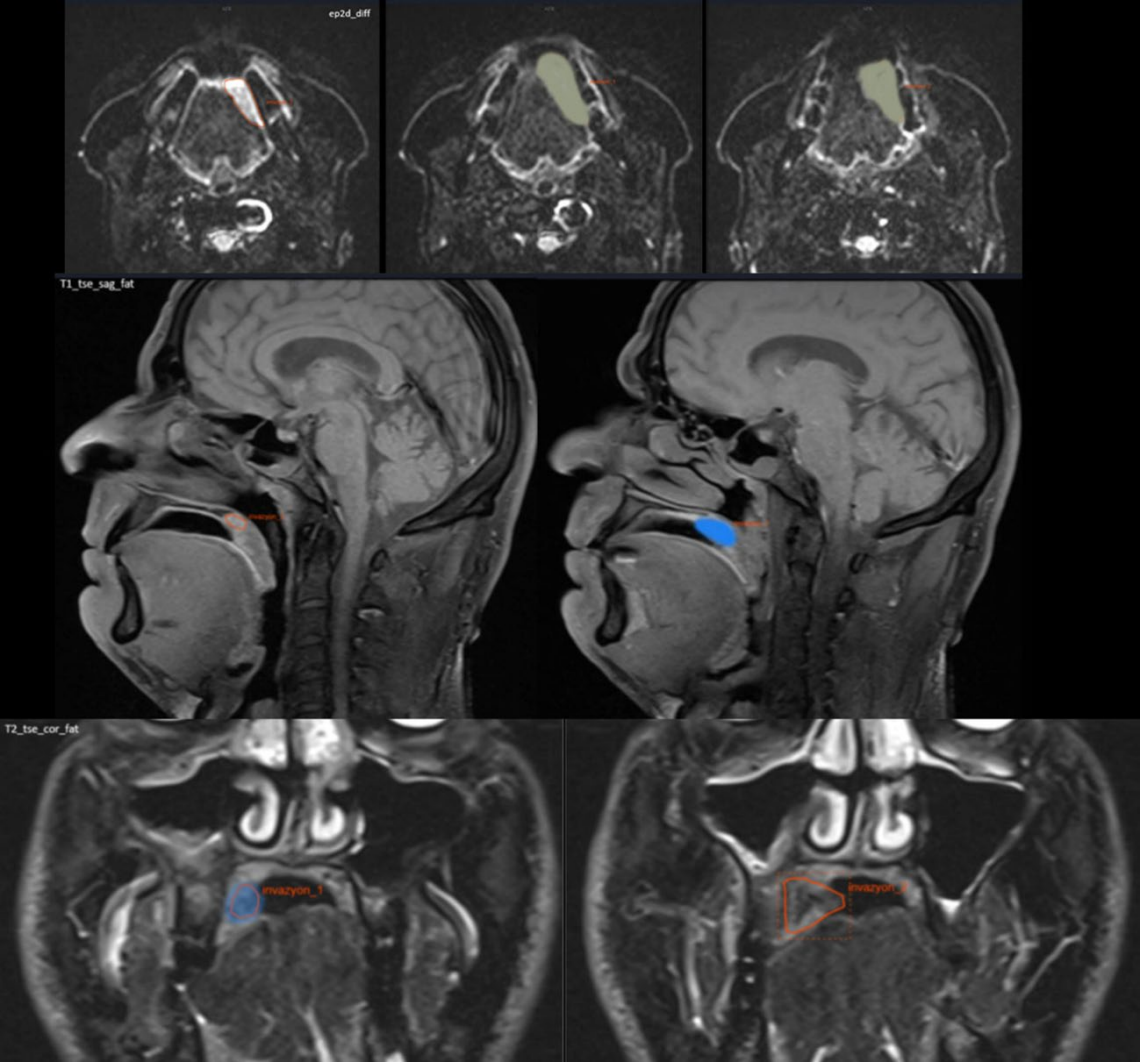
MR showing the segmentation of bone invasion using SVM ML model with manual segmentation and AI segmentation on axial, sagittal and coronal MR images with different sequences

Table 6 also shows the details of Confusion Matrix for detection of bone invasion using the highest learning classifier MLP Classifier (SVM). Figure 9 displays the manual and automatic AI segmentation and comparison of the segmentation using different slices and sequences of MR images.

The subsites and stages of the OSCCs analyzed in this study are detailed in Table 7, providing a comprehensive breakdown of the tumor locations and their classifications (T4a, T4b, and T4c). Additionally, Table 8 lists the types and anatomical locations of the benign lesions assessed in this study. These histopathological diagnoses and their specific sites within the oral cavity contribute to the overall evaluation of the model’s performance across a diverse set of lesion types, further validating its robustness and applicability in clinical settings.

**Table 6:** The details of confusion matrix in detection of bone invasion using SVM

Bone Invasion	SWM		
	True	False	Accuracy (%)
0	20	1	95
1	25	2	92
Accuracy (%)	45	3	93,4

0 indicates bone invasion free, 1 indicates invasion

**Table 7:** OSCCs and their locations, subsites, and staging (T4a-T4b-T4c)

		Oral Cancer Subsite						
		Lips	Alveolar Ridge	Hard Palate	Anterior 2/3 of the Tongue	Floor of Mouth	Retromolar Trigone	Buccal Mucosa
AJCC Staging	Stage IVA	1	2	1	11	10	5	5
	Stage IVB	0	1	1	1	1	1	0
	Stage IVC	0	0	0	1	1	0	0

**Table 8:** Types of benign lesions and their locations in the oral cavity

	Gingiva	Buccal Mucosa	Submandibular Gland	Tongue	Floor of Mouth	Parotid Gland	Parotid Gland	Lips	Retromolar Trigone	Hard Palate	Total
Pleomorphic Adenoma	0	0	6	0	0	0	0	0	0	1	7
Traumatic Fibroma	0	3	0	2	0	0	0	0	1	0	6
Pyogenic Granuloma	5	0	0	0	0	0	0	1	0	0	6
Peripheral Giant Cell Granuloma	3	0	0	0	0	0	0	0	0	0	3
Hemangioma	0	2	0	0	0	0	0	1	0	0	3
Warthin Tumour	0	0	0	0	0	3	3	0	0	0	3
Lipoma	0	2	0	0	0	0	0	0	0	0	2
Papilloma	0	1	0	1	0	0	0	0	0	0	2
Peripheral Ossifying Fibroma	2	0	0	0	0	0	0	0	0	0	2
Neurofibroma	0	1	0	1	0	0	0	0	0	0	2
Simple Ranula	0	0	0	0	2	0	0	0	0	0	2
Plunging Ranula	0	0	0	0	1	0	0	0	0	0	1
Schwannoma	0	0	0	1	0	0	0	0	0	0	1
Lymphangioma	0	0	0	1	0	0	0	0	0	0	1
Dermoid Cyst	0	0	0	0	1	0	0	0	0	0	1
Extra-osseous Ameloblastoma	1	0	0	0	0	0	0	0	0	0	1
Giant Cell Fibroma	1	0	0	0	0	0	0	0	0	0	1
Total	12	9	6	6	4	3	3	2	1	1	44

The performance of radiologists in diagnosing bone invasion in OSCC using MRI was also assessed. Radiologists demonstrated a sensitivity of 94% and a specificity of 100%. These values are within the range reported in the literature, where sensitivity for MRI in detecting bone invasion varies between 58.3 and 95.24%, and specificity ranges from 73 to 100%. High sensitivity is crucial to ensure most cases of bone invasion are correctly identified, while high specificity helps minimize false positives, aligning with clinical standards and supporting accurate diagnosis and effective treatment planning.

## Discussion

OSCC often infiltrates the mandible rapidly, impacting prognosis significantly. MRI stands out for its sensitivity and accuracy in evaluating bone invasion, soft tissues, and neurovascular infiltration, especially for diagnosing small lesions. However, while radiological images provide essential insights, they offer limited information about tissue complexity, prognosis, and treatment response. Leveraging radiomics—analyzing radiological quantities alongside clinical/laboratory parameters—proves crucial in unraveling survival rates, treatment responses, and disease morbidity.

Surprisingly, there’s a shortage of ML methods using MRI-based radiomics in the oral and maxillofacial region. Our study breaks ground as the first to propose an ML model using MRI-based radiomic analysis, aiming to detect OSCC and predict potential bone metastasis.

In the study by Mukherjee et al. [31], to determine the performance of CT-based radiomic features for predicting histopathological features of head and neck SCC, the study group showed moderate performance (AUC; 0.71, 0.75, 0.77, respectively) in predicting HPV status, tumor grade, and extracapsular invasion. It showed a low ability to predict perineural invasion and lymphovascular invasion (AUC; 0.64 and 0.69, respectively).

Corti et al. [21] developed an MRI-based radiomic signature to improve prognostic prediction and overall survival in OSCC on a multicenter, retrospective dataset (*n* = 123), and validated it in a prospective cohort (*n* = 108). This radiomic signature demonstrated significant prognostic power for overall survival.

In the study by Wes et al. [20], to improve and validate MRI-based radiomic prognostic models in oral and oropharyngeal cancer, the most accurate models were identified by integrating radiomic and clinical variables. The AUC was 0.72 (survival) and 0.74 (survival without recurrence) for oral cancers and 0.81 (survival) and 0.78 (survival without recurrence) for oropharyngeal cancers and concluded that MRI radiomics provides additional prognostic information to known clinical variables, with the best performance of the combined models.

Bos et al. [32] validated a pretreatment MRI-based radiomics model for predicting locoregional control in oropharyngeal squamous cell carcinoma and evaluated the impact of differences between datasets on predictive performance in a group of 157 patients. Their study concluded that the radiomics model had an AUC of 0.74, sensitivity/specificity of 0.75/0.60, and an accuracy of 0.71.

In the present study, we proposed an ML model with radiomics analysis using MRI images, similar to the work of Wes et al. [20], Corti et al. [21], and Bos et al. [32] and examined the ability of this model to detect OSCC and predict possible bone metastasis. The ability to predict bone metastasis (AUC = 0.999) was found to be excellent. In this respect, our study has quite different results from other studies in the literature.

It is possible with a data-driven approach to extract meaning from certain clinical symptoms, and radiological images and teach the computer what they look like using iterative algorithms and ML. With ML, it is possible to classify tumors and detect lesions [33]. There are a limited number of studies in the literature that use an ML model with CT and MRI-based radiomic analysis.

Guo et al. [34] evaluated the potential of CT-based radiomic features to predict thyroid cartilage invasion in patients with laryngeal and hypopharyngeal squamous cell carcinoma using 86 patients with thyroid cartilage invasion and 179 patients without invasion. LR and LR-SVMSMOTE were used as ML models and they found the AUC to be 0.876 and 0.905, respectively. The results showed that CT-based radiomic features have great potential with a satisfactory prediction performance of thyroid cartilage invasion.

In the study conducted by Park et al. [23], to predict pathologic factors and treatment outcomes of oropharyngeal SCC patients using ML and radiomic features obtained from MRI images of 155 preoperative patients, the AUC values of the LR and LightGBM model were 0.792 and 0.833, respectively.

Yuan et al. [24] used LR, RF, naive Bayes (NB), SVM, AdaBoost, and neural network (NN) ML models to predict occult cervical lymph node metastasis in early-stage oral tongue SCC from MRI tissue features and to develop and compare various ML models. NB model gave the best overall performance, correctly classifying the nodal state in 74.1% (86/116) of carcinomas with an AUC of 0.802. NB also had the highest values for accuracy, sensitivity, specificity, F1, precision, and recall.

Unlike other studies in the literature, 6 ML methods, namely NN, SVM, XGBoost, RF, LR, and DT, were utilized in this study. The SVM method has the highest AUC, precision, sensitivity, specificity, F1, precision, and recall values.

While our study marked a new effort in its field, it encountered several limitations. This was a retrospective analysis with a relatively small cohort of 86 patients: 44 benign cases without bone invasion and 42 malignant cases with bone invasion. Our reliance solely on MRI imaging restricted our assessment to bone invasion alone.

In future investigations, we aim to expand our scope. This includes evaluating HPV status, tumor grade, lymphovascular invasion, extracapsular invasion, and more, leveraging radiomics models and ML techniques. This future research will encompass larger, more diverse populations and integrate various imaging methods such as CT and cone-beam CT for a comprehensive analysis.

## Conclusion

In summary, our study underscores the potential of MRI-based radiomics aided by machine learning in early OSCC detection and bone metastasis prediction, showcasing an exceptional AUC of 0.999. The integration of radiomic features from MRI offers insights into identifying and characterizing bone invasion in OSCC, aligning with previous research in head and neck malignancies. Despite our study’s strengths, particularly the robust performance of the developed model, limitations such as a retrospective design and a relatively small sample size underscore the need for future investigations. Expanding research parameters to include HPV status, tumor grade, and diverse imaging modalities like CT scan augment our understanding of OSCC’s metastatic behavior.

This research forms a foundation for future studies, supporting for the incorporation of advanced imaging techniques, radiomics, and ML in clinical settings. Larger-scale studies about diverse parameters and imaging tools are imperative, promising enhanced OSCC diagnosis, prognosis, and personalized patient care.

## Abbreviations

ML: Machine-learning
AI: Artificial Intelligence
MRI: Magnetic Resonance Imaging
CT: Computed Tomography
IARC: The International Agency for Research on Cancer
T1-SE/TSE: T1-weighted Turbo Spin Echo
T2-TSE/FSE: T2-weighted Turbo Spin Echo/Fast Spin Echo:
T1-FS: T1-weighted fat-saturated
T2-FS: T2-weighted fat-saturated
DWI: Diffusion-weighted Imaging
ETL: Echo Train Length
CET1W: Contrast-enhanced T1-weighted
GLCM: Gray Level Co-Occurrence Matrix
GLRLM: Gray Level Run Length Matrix
GLSZM: Gray Level Size Zone Matrix
IBSI: Imaging Biomarker Standardization Initiative
LR: Logistic Regression
RF: Random Forest
DT: Decision Tree
KNN: K-Nearest Neighbors
SVM: Support Vector Machine
NB: Naive Bayes
NN: Neural Network
